# Experiences and coping behaviours of clients with sexually transmitted infections in Malawi

**DOI:** 10.4102/hsag.v31i0.3131

**Published:** 2026-04-29

**Authors:** Thandie Munthali, Chimwemwe Munthali, George Chapweteka, Patson Kumwenda, Nixon Chidzere, Catherine Mwale, Esmie Mkwinda, Geldine Chironda

**Affiliations:** 1 Department of Clinical Medicine, Faculty of Health Science, Saint John of God University, Mzuzu, Malawi; 2 Faculty of Health Sciences, Mzuzu University, Mzuzu, Malawi; 3 Department of Nursing and Midwifery, Faculty of Health Sciences, Saint John of God University, Mzuzu, Malawi

**Keywords:** lived experiences, coping behaviours, client, sexually transmitted infections, Primary Health Centre, Malawi

## Abstract

**Background:**

Sexually transmitted infections (STIs) remain a significant public health challenge in Malawi, affecting millions of people every year. Despite the availability of effective treatments, STIs can have a significant impact on the well-being of those infected.

**Aim:**

The study aimed at exploring the lived experiences and coping behaviours of clients attending the STIs clinic at Mzuzu Urban Health Centre.

**Setting:**

Urban Primary Health Centre in Mzuzu.

**Methods:**

A qualitative approach with a descriptive phenomenological design was used in this study. Ten participants were purposively selected. A semi-structured interview guide was used to collect information on demographics, experiences and coping behaviours. Data collected was thematically analysed.

**Results:**

Lived experiences were emotional strain on relationships, feelings of fear, Stigma and discrimination from the community, uncertainty about the future, anxiety and depression symptoms of the disease. However, adaptive coping behaviours were to seek comfort in close family members and friends and intrinsic personal motivation. While maladaptive coping behaviours were utilising traditional practices on the genitalia, and avoidance behaviours.

**Conclusion:**

Individuals with STIs predominantly face psychological experiences, concerns about fertility, with economic worries, often worsened by the link to HIV. Understanding the experiences and their coping strategies can help healthcare providers to intensify public awareness and health education on the treatment of STIs. Additionally, integration of mental health interventions for more personalised, holistic care is crucial.

**Contribution:**

The study highlights the psychological burden experienced by clients with STIs and importance of public awareness on treatment.

## Introduction

Sexually transmitted infections (STIs) represent a significant public health and social challenge worldwide, especially in developing countries (Caruso et al. 2021). According to Feroli and Burstein (2003), STIs can be transmitted through various sexual activities, including during pregnancy, childbirth and breastfeeding. Some STIs may be asymptomatic, while others show symptoms in the genital area. The eight most common STIs include four curable infections (chlamydia, gonorrhoea, syphilis and trichomoniasis) and four treatable but incurable infections (Hepatitis B, Herpes simplex virus, HIV and Human Papillomavirus [HPV]) (Caruso et al. 2021). Johnson et al. (2011) noted that STI control in low- and middle-income countries is primarily guided by clinical case management guidelines, which emphasise syndromic management.

The World Health Organization (WHO 2021) reported approximately 376 million new cases of STIs globally in 2019, with the highest incidence occurring in young people aged 15–24 years. The regions with the highest rates of STIs are Sub-Saharan Africa and South and Southeast Asia, with Sub-Saharan Africa having a particularly high prevalence of HIV as one of the major STIs, affecting an estimated 25.6 million people in 2019. In this region, the HIV prevalence among adults aged 15–49 years is around 2.5%, with a higher rate of 13.5% in some areas (WHO 2021). In Malawi, the HIV prevalence among adults aged 15–64 years was estimated at 10.6% in 2019 (Payne et al. 2023), and this translates to a high burden of STIs in the country. Factors contributing to the high prevalence of STIs in Sub-Saharan Africa and Malawi include limited healthcare access, poverty, lack of comprehensive sex education and stigma. If not properly treated, STIs can cause severe complications, including cervical cancer, chronic genital pain, infertility, pelvic inflammatory disease and increased HIV transmission (Van Gerwen, Muzny & Marrazzo 2022).

Effective management of STIs involves timely diagnosis, appropriate medication, partner notification, safe sexual practices, vaccinations (where available), regular screenings and education (Van Gerwen et al. 2022). Treatment for STIs typically involves a combination of medications, such as antibiotics, antifungals and antivirals, alongside lifestyle changes and preventive measures, depending on the specific STI diagnosed (Gannon-Loew & Holland-Hall 2020). Healthcare professionals specialising in sexual health, as well as primary care providers like nurses, clinical officers and gynaecologists, play crucial roles in diagnosing and treating both simple and complex STI cases (Barrow 2020). Primary healthcare centres offer basic STI services, district hospitals provide more comprehensive care, while central hospitals and specialised STI clinics handle complex cases and offer in-depth diagnostic, treatment and prevention services. The government of Malawi, in partnership with international organisations and stakeholders, has set strategic goals to promote sustainable development in the prevention, control and management of STIs (WHO 2021).

However, individuals seeking care at STI primary healthcare clinics often experience psychological distress, which can vary based on their circumstances and reasons for seeking treatment (Brewer et al. 2020). Psychological distress, characterised by symptoms of depression and anxiety, is a common mental health issue (Townsend & Morgan 2017). Certain groups, such as newly diagnosed STI patients, their partners, individuals with a history of childhood sexual abuse, sex workers and Lesbian, gay, bisexual, transgender, queer (and more) (LGBTQ+) individuals, may be more vulnerable to such distress. Additionally, people with STIs face not only physical health challenges but also psychological burdens related to stigma and discrimination (Lee & Cody 2020).

Individuals diagnosed with STIs adopt a range of coping behaviours that can either alleviate or worsen psychological distress (Craig et al. 2019). Some of the reported strategies include seeking medical care and emotional support (McCormack & Koons 2019; Mehta et al. 2024). However, researchers have reported harmful coping strategies, which include substance use, risky sexual behaviours, which are influenced by social networks (Evers et al. 2020). Singh and Singh (2021) highlight the psychological burden STIs can place on self-concept and mental health, emphasising the need for tailored psychological support. In Malawi, primary healthcare centres offer basic STI services that give in-depth diagnostic, treatment and prevention services. However, there is minimal literature on the lived experiences and coping behaviours of clients with STIs attending the primary health care centres in Malawi.

## Research methods and design

### Research design and setting

This study employed a descriptive phenomenological design to explore the lived experiences and coping behaviours of clients with STIs. The study took place at Urban Primary Health Centre, located in Mzuzu, one of the three cities in northern Malawi. As one of the busiest community primary healthcare centres in the country, it receives referral patients from 23 small health centres, which suggests that the findings may provide an accurate representation of the situation in Mzimba North.

### Study population and sampling strategy

The study population consisted of clients who sought treatment and medication at the STI clinic, which is within the Outpatient Department (OBP) of Mzuzu Urban Health Centre. This hospital was selected because it provides health services to a catchment population of about 6000 per month, with an expected 2000 STI clients per month, with an average of 200 adult OPD patients daily. Patients with STIs are typically frequent visitors. As determined by data saturation, the sample of 10 clients who attended the STI clinic was selected using a convenience sampling method. Convenient sampling was ideal in the Malawian context because the STI clinics are run independently and therefore, easily convenient to find these participants. The following were requirements for inclusion: men and women 18 years and older who attended the STI clinic for, treatment and counselling.

### Data collection

The participants were interviewed using a self-developed in-depth semi-structured interview guide with open-ended questions. The first part of the guide was used to collect the personal characteristics of participants, like patients’ age, sex, occupation, education, socioeconomic status and marital status. The second and last part was used to elicit information on psychological experiences and coping behaviours of STI clients. The tool was translated into the Tumbuka language. Data was collected for 1 month in March 2024, from Monday to Friday, since the STI clinic operates on weekdays. On the first day, the researcher sought permission from the representative of the Director of Health and Social Services (DHSS) the District Medical Officer (DMO), about the intention of conducting data collection from the participants attending the STI clinic. The DHSS or the DMO directed the researcher to the adult outpatient department’s clinical in charge.

Thereafter, the clinical in-charge briefed the OPD clinicians and nurses about the data collection activity.

Before giving the research tool to each study participant, the researcher explained the aim of the study and the ethical issues surrounding the study to the participant. The participants who were interested in taking part in the study were asked to sign an informed consent form voluntarily. After signing the consent form, interviews were recorded using a tape to avoid missing some information and self-administered questionnaires were distributed to the participants who were literate. In addition, some notes were written down, including non-verbal ones, to establish the trustworthiness of the data. These interviews were held in a private room within the STI department during the first quarter of March 2025, with each interview lasting between 25 min and 45 min.

### Data analysis

The thematic analysis approach was used to analyse data. Sundler et al. (2019) emphasised descriptive phenomenology as a specific approach within thematic analysis that focuses on the description and interpretation of individuals’ lived experiences. The researchers applied Braun and Clarke’s six-step inductive thematic data analysis approach (Braun & Clarke 2006). The first step involved the researcher reading and transcribing the data to become thoroughly acquainted with it and making initial observations. In the second step, the researcher broke down the data into meaningful segments and assigned codes that captured the essence of each part. The third step involved grouping the codes into broader themes to identify emerging patterns or ideas. The fourth step required the researcher to refine and validate the themes by comparing them to the data to ensure their accuracy and relevance. In the fifth step, each theme was clearly defined and assigned a name that represented its core meaning. The final step involved presenting the findings using data excerpts to support the identified themes and discussing their broader implications.

### Trustworthiness

The researcher ensured the trustworthiness of qualitative data in terms of credibility, dependability, conformability and transferability (Lincoln & Guba 1986). Extended, prolonged interactions with participants, lasting between 25 min and 45 min, were essential for establishing credibility. Member checks with study participants were employed in this study to confirm that the data accurately represented their experiences. To ensure that the interpretations and conclusions aligned with the transcript analysis, the researchers also consulted an independent coder who confirmed the themes. However, there was a slight difference in the naming of subthemes, which was resolved through discussion with the research team. Transferability was assured by providing a detailed explanation of the study’s participants, methodology and context. Direct quotes from participants were incorporated to support each theme and subtheme, reinforcing the authenticity of the findings.

### Ethical considerations

Ethical clearance to conduct this study was obtained from the Research Ethics Committee at Mzuzu University. The ethical clearance number is FOHS/STJOG/24/007. Permission to conduct the research was granted by the Mzimba North District Health Office (DHO) through the DMO/Research Committee. After the study’s objectives and participant expectations were fully explained, all participants provided written consent. The interviews were conducted in a prearranged private room at the Mzuzu Health Centre, and codes were used to maintain anonymity. Participants were assured that all data related to the study, including personal information, would be kept confidential. The transcripts and digital recordings will be securely stored until the research objectives have been met.

## Results

### Characteristics of the study participants

The sample had 10 participants. Their age ranges from 18 to 50 years. The sample comprised four males and six females. There were two students, while six were married and two were single (Table 1).

**TABLE 1:** Characteristics of study participants.

Participant	Age range (years)	Marital status	Religion	Education	Tribe
P1	18–22	Single	Christian	Secondary level(student)	Chewa
P2	22–25	Single	Christian	Secondary Certificate level	Chewa
P3	22–25	Single	Christian	Junior Certificate level	Tumbuka
P4	22–25	Married	Christian	Tertiary level	Tumbuka
P5	30–35	Single	Christian	Tertiary level	Tumbuka
P6	30–35	Married	Christian	Tertiary level	Yao
P7	30–35	Married	Christian	Primary level (dropped out)	Yao
P8	40–45	Married	Muslim	Junior Certificate level	Chewa
P9	40–45	Married	Christian	Junior Certificate level	Tumbuka
P10	50	Married	Christian	Junior Certificate level	Tumbuka

### Experiences and coping behaviours of clients at the sexually transmitted infection clinic at Mzuzu Health Centre

The key themes that developed were: Experience, adaptive and maladaptive coping behaviours. The subthemes that described experiences were: Emotional strain on relationships, feelings of fear, stigma and discrimination from the community, anxiety and depressive symptoms of the disease, and uncertainty about the future (Table 2). The subthemes for adaptive coping behaviours were: Seeking comfort in close family members and friends, and intrinsic motivation. While those for maladaptive coping behaviours were: utilising traditional practices on the genitalia, avoidance behaviours (Table 2). Each of these is described in detail below.

**TABLE 2:** Experiences and coping behaviours of clients attending sexually transmitted infection clinic.

Key theme	Subthemes
Experiences	Emotional strain on the relationships
	Feelings of fear
	Stigma and discrimination from the community
	Uncertainty about the future
	Anxiety and depression are symptoms of the disease
Adaptive coping behaviours	Seeking comfort in close family members and friends
	Intrinsic personal motivation
Maladaptive coping behaviours	Utilising traditional practices on the genitalia
	Avoidance behaviour

### Description of the subthemes on the experiences of clients attending the sexually transmitted infection clinic

#### Emotional strain on relationships

Participants reported that STIs caused emotional strain in their relationships, leading to issues with intimacy, feelings of guilt and fears of abandonment by their partners because of perceived risks and emotional consequences. These struggles were reflected in the challenges couples faced in their intimate connections. The study results indicated that emotional strain significantly impacted both individual well-being and relationship dynamics, resulting in intimacy issues, emotional distress and potential relationship breakdowns. This is expressed in the following quotes:

‘It affected me so much because I thought my relationship could end.’ (Participant 8)

Another participant added the burden experienced by their partners after denying them sex:

‘It is a burden as a couple because you don’t feel like having sex with your partner, and when he wants to sleep with you, it’s worrisome because of the pain when you deny him sex; it’s also a burden to him.’ (Participant 10)

#### Feelings of fear

Fear is a prevalent emotion among individuals diagnosed with STIs. The findings reflect the anxiety and fear associated with the illness, not only about the immediate impact but also concerning the potential future outcomes. For example, phrases like:

‘I thought this was the end of my life because I’m already HIV positive.’ (Participant 9)

Additionally, the fear of physical pain and the progression of the disease, such as ‘when he wants to sleep with you, it’s worrisome because of the pain’, illustrates the combination of fear of physical discomfort and emotional distress. These expressions underscore the profound uncertainty and fear that come with an STI diagnosis. This fear of the unknown contributes to emotional distress:

‘I was worried because people said this problem can kill me because the symptoms start inside and then manifest outside the body.’ (Participant 1)

#### Stigma and discrimination from the community

These quotes highlight the intersection of socioeconomic status, stigma and STIs. The fear of being judged because of the disease is clearly a source of additional emotional stress. The worry about others’ assumptions regarding one’s sexual behaviour further compounds the anxiety and emotional turmoil. The study has revealed that several clients mentioned concerns about being judged by others, especially regarding the misconception that STIs signify promiscuity or HIV status. This is a powerful source of psychological distress linked to fear of rejection or misunderstanding by society. Stigma and discrimination are recurring themes, as illustrated by phrases such as:

‘“It was very painful because I’m already a poor person and I was worried that maybe I have contracted HIV,” and “People may think I have multiple partners and HIV, yet I don’t have.”’ (Participant 4)

One client said, ‘It affected me so much because people may think I have multiple partners and HIV because HIV and STIs go hand in hand.’ (Participant 3)

#### Uncertainty about the future

The study has revealed that many participants report fearing for their future health, relationships and fertility, with some worried that their condition might lead to the end of their relationships or their ability to have children. These individuals grapple with fears about their relationships, personal fulfilment and societal perceptions. It is a powerful expression of the stress that accompanies the disease’s potential to disrupt their lives, dreams and the concept of normality. For example, this quote:

‘I was not happy because I thought I would not get married and not be able to have children.’ (Participant 9)

The study has shown that for those who are already HIV-positive, the STI compounds the fear about the long-term effects on their health, relationships and future. This group is particularly concerned with the potential worsening of their condition or the end of their relationships:

‘It affected me so much because I contracted HIV from my husband … how will my health, future, and marriage be like?’ (Participant 7)

#### Anxiety and depression are symptoms of the disease

The emotional toll of STIs is significant with many individuals reporting symptoms of depression and anxiety. reveals the confusion and isolation felt at the onset of symptoms. It also highlights the psychological burden of not understanding one’s condition. The feeling of hopelessness is emphasised some quotes: All this pointed to the emotional struggle in trying to find solutions to the disease, only to face worsening health issues. Such emotional pain is central to the lived experience of many STI-positive individuals. This is expressed in the following quotes:

‘I was depressed because I didn’t know what was happening to me, and my aunt thought I was seeing someone else.’ (Participant 5)

‘I feel sad because I slept with a girl hoping that my problem would end, but it increased.’ (Participant 2)

#### Description of the subthemes for coping behaviours

The identified coping behaviours were adaptive (Seeking comfort in close family members and friends; Intrinsic motivation) and maladaptive (utilising traditional practices on the genitalia; avoidance behaviours). Each coping behaviour is described in detail below:

#### Seeking comfort in close family members and friends

The study has shown that family and friends are key sources of emotional strength for clients. Encouragement from family members, particularly mothers and spouses, provides significant motivation, while friends offer comfort and a sense of normalcy through social interactions. This highlights the importance of a supportive network in helping individuals navigate the emotional challenges of STI management:

‘My mother, a village leader, encouraged me so much.’ (Participant 1)

‘I was always surrounded by friends and chatting as a source of comfort and engaging in small-scale businesses.’ (Participant 9)

#### Utilising traditional practices on the genitalia

The findings show that some clients practised self-care strategies, including both modern and traditional approaches to manage the symptoms of STIs or well-being. This includes practices like using cold water or steam treatments, which indicate the integration of cultural beliefs alongside modern medical care, highlighting the importance of culturally sensitive health interventions:

‘I used cold water to wash my genitalia to find relief from the pain as part of a traditional self-care practice.’ (Participant 7)

‘I was using steam from traditional medicine to warm their genitalia as a form of treatment.’ (Participant 8)

#### Intrinsic personal motivation

The findings of the study have also revealed that some clients used personal encouragement as a way of motivating themselves to cope with the STI. This internal drive is essential, as individuals actively encourage themselves to take steps toward improving their health, such as seeking medical attention or maintaining a positive outlook. The study has shown that some clients stay distracted from their problems or illness by focusing on daily tasks, work or school to avoid dwelling on their illness or issues, keeping their minds engaged. This not only helps to distract them from the stress of their health concerns but also gives them a sense of purpose and normalcy. The ability to concentrate on daily tasks fosters resilience and provides a constructive outlet for emotional energy. This is all expressed in the following excerpts:

‘I encouraged myself to visit the hospital and seek help.’ (Participant 2)

‘I focused on working as a carpenter, and self-encouragement helped them avoid thinking about the disease.’ (Participant 4)

‘I concentrated much on my school and studying was a helpful distraction.’ (Participant 3)

#### Avoidance behaviour

The study has revealed that some clients engage in certain behaviours to avoid situations that may worsen their condition, e.g. avoiding sexual intercourse. This demonstrates the proactive steps individuals take in managing their health and reducing the risk of transmitting the infection to others:

‘I started refusing to sleep with my husband to prevent escalating my condition.’ (Participant 10)

## Discussion of the findings

The study explored the experiences and coping behaviours of clients with STIs in an STI clinic in one of the urban community hospitals, northern Malawi. Ten clients diagnosed with STIs were interviewed, and their ages ranged between 18 and 50. This age group is regarded as a sexually active group in the Malawian context, indicating an at-risk age group for contracting STIs. Out of the 10 clients that were interviewed, six were females. Even though these clients were conveniently identified, this probably implies that women seek STI services more as compared to men.

On the other hand, out of 10, six clients were married, which would indicate infidelity among those married. However, this may also indicate that those who are married seek STI services more as compared to their counterparts. These results have similarities with the earlier study done in Bangladesh by Huda et al. (2022:1906), who found 10% of ever-married women aged 15–49 years had abnormal genital discharge and genital sores/ulcers among nevertheless, women aged 25–34 years who used contraceptives and married earlier had an increased likelihood of STI symptoms. Nevertheless, limited choices in sexual decisions among married individuals and the inability to abstain from sexual intercourse with a cheating spouse have been reported as a risk to contracting STIs (Pintea-Trifu et al. 2024:1449). On the contrary, Zaki et al. (2021), in their study, found that unmarried women in Lebanon had a greater risk of contracting STIs because of the discontinuous use of condoms and trust in their partners. Although the two studies used different methodologies and assessed different variables, health-seeking behaviour and exposure to STIs are portrayed more among women, while the risk between married and unmarried remains controversial.

Further, researchers have reported the role of religion in health-seeking behaviour among clients with STIs. According to Sawyerr (2019), religion was found to be a significant predictor of health-seeking behaviours among clients with STIs. The study by Banda Kamanga et al. (2023) found that prayers have a prominent role in the management of STIs and HIV. However, this may create barriers to health-seeking behaviours among clients with STIs, especially in Malawi, where Christianity and cultural beliefs coexist. Contrary to this, religion has been reported to significantly contribute to increasing knowledge on prevention of STIs and HIV through organised awareness programmes, as indicated by Njeula (2022).

In this study, the authors found that clients attending the STI clinic experience a wide range of psychological experiences. The experiences were related to emotional strain on relationships, feelings of fear, stigma and discrimination from the community, anxiety and depressive symptoms about the disease and uncertainty about the future. Clients reported that STIs affected their intimacy and connection within relationships, leading to emotional and physical strain within the couple. Because of discomfort and pain during sexual intercourse, clients avoided sex with their spouses, which not only affected marital relationships but also induced fear of the marriage ending.

These findings are similar to earlier studies, which revealed novel insights into mental health implications associated with STIs (Bretz, Keshwani & Raphael 2023:134; Pintea-Trifu et al. 2024:1449; Scheinfeld 2021:7179). While individuals with the STI had lower adaptability in couples where at least one partner harbors suspicion of an STI, those with the STI exhibited a heightened level of anxiety at the moment. On the other hand, a study done in India by Singh and Singh (2021) noted specific STIs, such as genital herpes and warts had indicated significantly more overall depression, cognitive impairment and stress as compared to the patients suffering from syphilis.

Similarly, emotional burdens like guilt and worries about relationships ending created a significant gap in the relationship dynamic. While in this study, the authors did not look into specific STIs, emotional experiences are similar, and this may indicate emotional and mental dejection to those affected by STIs (Bretz et al. 2023:115–134). The study findings highlight the importance of addressing the emotional and psychological challenges through conducting personal and private discussions with patients to address their fears and questions and integrating mental health interventions to support STI clients (Kirschnick et al. 2024:990–1001).

Likewise, in this study, clients feared that an STI diagnosis could be a life-threatening condition that could lead to severe health consequences like HIV-related complications. This affected the client’s mental well-being because ofuncertainty. Studies have reported perceived fear of STIs as potentially life-threatening because oftheir association with HIV and other serious health outcomes (Kontomanolis et al. 2017:111–118). This perception can lead to a cycle of anxiety where clients may avoid seeking treatment for fear of their diagnosis or judgment from health workers and society at large. Consequently, this avoidance behaviour can exacerbate health risks and hinder effective management of STIs (Workowski & Bachmann 2022:S89–S94)

Clients also reported feelings of depression because of their STI diagnosis, especially when they believed that their condition was a terminal situation. Participants also experienced frustration, confusion, hopelessness and sadness because of misinterpretations of their condition by partners, family members and friends. This sense of despair can be exacerbated by engaging in sexual activity, which may only worsen their problems. These findings are similar to a study done by Tabata (2018), which found that clients articulated their experiences with depression and confusion following an STI diagnosis. These findings provide insight into how these emotions may manifest in clients dealing with STIs, emphasising the importance of mental health support to help individuals process their feelings after the diagnosis news. Addressing these emotional challenges is essential for improving the overall well-being of clients diagnosed with STIs. Again, this shows a need for supportive counselling and integration of mental health services to address mental health aspects of clients diagnosed with STIs (Bretz et al. 2023).

Furthermore, some clients expressed concerns about being perceived as ‘vulnerable’ or ‘diseased’ because ofboth their health status and socio-economic position. This suggests a broader social context where individuals feel judged both for their health and their socio-economic status. A study done by Aryal (2017) revealed significant concerns among clients regarding their health and socio-economic position. These concerns are rooted in the fear of being perceived as ‘vulnerable’ or diseased, which can lead to feelings of judgment and stigma from society.

Similarly, Aloh et al. (2020) in their study reported that socio-economic factors significantly influenced access to healthcare services, quality of care received and overall health outcomes. Clients’ socio-economic struggles may overshadow their health needs, leading healthcare providers to make assumptions about their lifestyle choices or commitment to treatment. This dynamic may create a barrier to effective communication between clients and providers, potentially compromising the quality of care. Addressing these socio-economic disparities is essential for improving health outcomes and ensuring equitable access to health services.

Nonetheless, some participants feared that their STI would prevent them from achieving life goals, particularly getting married and having children. The study revealed the uncertainty that participants feel about their prospects, as they fear that their condition will lead to the loss of these important milestones, especially from fertility challenges. Studies by Nganzo (2022) and Jansson et al. (2023), established worries about the long-term impact of their STIs on health, relationships and potential disclosure, which can create trust issues and emotional distress in romantic relationships. Expressing doubts about marrying and the ability to have children affected those diagnosed with STIs (Nganzo 2022). Nevertheless, clients expressed concerns about the long-term effects of their STIs on both their health and relationships. Additionally, there is a recurring sense of uncertainty about what the future holds in terms of health outcomes, relationships and personal well-being.

Another study reported frequent worry about how to disclose STI status and fear of negative impact on romantic relationships or prospects for marriage (Jansson et al. 2023). This apprehension stems from fears about how such disclosure might impact their current or future relationships. Concerns about transmitting an infection to a partner and potential for a relationship breakup because oftrust issues can lead to significant emotional distress (Jansson et al. 2023). While trust issues arising from STI disclosure can further complicate relationship dynamics, potentially resulting in breakdowns in communication and intimacy. Bretz et al. (2023) highlights the importance of mental health support to address feelings of despair.

The study also found that clients employed various coping behaviours which were both adaptive and maladaptive. Adaptive coping behaviours, such as sharing their stress or concerns with friends and family members, helped them to cope with the situation. Similarly, intrinsic personal motivation, seeking help and a positive sense of self assisted them in coping with the diagnosis.

Other participants reported that they got relief through sharing with close family members and friends. Sharing played a crucial role in providing emotional reassurance and motivation to clients. These interactions offered emotional relief, reduced isolation, and fostered a sense of normalcy and belonging, which is particularly important for clients dealing with the stigma of STIs. Support networks have been reported to play a crucial role in coping with long-term health conditions like STIs (Gao et al. 2010; Obeagu & Obeagu 2024:1–16).

People living with HIV and AIDS and with strong social support are likely to manage their emotional distress and adhere more to treatment protocols compared to those with limited social support (Berg, Page & Øgård-Repål 2021; Coulson & Buchanan 2022; Obeagu & Obeagu 2024:1–16). Strengthening social support through health education and public awareness is important in mitigating mental health challenges associated with STIs.

Clients also reported using self-encouragement and motivation as a coping strategy. Self-motivation is a powerful tool for individuals as it helps individuals’ medical seeking behaviour and positive stay. Some reported using routine activities like school and work to divert their attention from the STI diagnosis. This helped them to keep their minds engaged, reduce stress and offer a sense of normalcy despite the emotional distress of living with an STI. Studies have reported that people with a higher intrinsic motivation adhere to treatment regimens for chronic conditions such as HIV better. This suggests a key role the intrinsic motivation plays in long-term health management and a need for counselling services to build intrinsic motivation among those affected. (Slemp et al. 2024).

On the other hand, participants reported utilising maladaptive coping behaviours, such as avoidance and utilising traditional practices on genitalia care. It was noted that the use of maladaptive coping behaviours was mainly because of fear of being known to have an STI diagnosis. Clients diagnosed with STIs are reported to be anxious that people might judge them as promiscuous or assume they have HIV as a result, they face social exclusion and judgment (Lariat et al. 2023; Than et al. 2019). This type of fear and emotional pain stemming from stigma can exacerbate feelings of shame and isolation. Nevertheless, avoidance behaviour can prevent them from accessing necessary medical care and support services, further compromising their mental well-being.

Some participants reported relying on traditional and physical self-care practices to alleviate symptoms or manage their condition. Even though these practices may have provided comfort however their safety remains questionable. The use of traditional practices may indicate a lack of awareness regarding STI treatment and complications that may come out of infection. Agbemavi (2021) in their study, also found that people with STIs use traditional herbal treatments to soothe genital sores or infections. Recent studies (Kacholi & Amir 2023; Mongalo & Raletsena 2022; Ngoben et al. 2023) put emphasis on cultural practice specific rituals such as herbal steam baths or the use of locally grown plants to manage genital health. Even though these cultural practices are seen as a form of self-care, offering a sense of empowerment and control, they may indicate fear of being stigmatised or a lack of health information. Earlier studies on reported lack of knowledge, fear of confidentiality of personal information, shame and stigma and lack of integration of services contributed to poor seeking care and acceptability of STI services (Newton-Levinson, Leichliter & Chandra-Mouli 2016). There is a need for public awareness on STI treatment and its complications.

Other clients used avoidance behaviours, such as refraining from sexual activities and discussing issues regarding STIs. The use of avoidance could imply a marital relationship and may hinder medical treatment-seeking behaviours in the long term. A study by Rueda et al. (2016) reported that individuals with HIV often engage in avoidance behaviour, including refraining from sexual intercourse, because of the stigma and fear of transmission, and emotional and psychological stress associated with their diagnosis. Even though avoidance can sometimes exacerbate relationship difficulties, it may also be perceived as a form of protection. Avoiding engaging in sexual activities could help in preventing further infection and minimise physical complications and the risk of transmitting the STI to others.

This study found that both coping behaviours played a critical role in managing the psychological and mental health impact of the clients. However, it should be cautioned that maladaptive behaviours may result in a long-term health impact, such as depression and other STI complications, especially to those who report using traditional practices on genitalia care. Similarly, coping behaviours can significantly influence clients’ psychological well-being and adherence to treatment (Dafesh 2017). Healthcare providers should recognise these coping behaviours when developing treatment plans and provide resources that enhance positive coping strategies while addressing any maladaptive behaviours. Furthermore, understanding these coping mechanisms is crucial for healthcare workers aiming to provide holistic care that addresses both physical and mental health needs of clients diagnosed with STIs.

### Strengths and limitations

The strength of the study lay in the ability of the participants to provide sensitive, rich and in-depth descriptions of their experiences with STIs. Thus, the participants were given the platform to share their sensitive issues in their own words. In terms of limitations, some data might have been selectively reported by participants, resulting in possible information bias. This might be possible because Malawian culture views STIs through the lens of stigma. Researchers’ experiences in mental health and STI might have introduced the researcher bias; however, this was mitigated by the researchers constantly reflecting on their understanding of the subject matter. Recall bias was evident as participants struggled to accurately remember past emotional responses and experiences with an STI, particularly those from a long time ago. However, this bias was mitigated through member checking, where the researcher contacted the participants to verify the given information. Further, peer debriefing was conducted with all research team members to validate the interpreted information.

## Conclusion

The study findings highlight that clients attending STI clinics face emotional challenges, including relationship strain, fear, stigma, anxiety and depression. STIs affect intimacy, leading to emotional and physical distress. Clients also fear being associated with HIV, which worsens stigma and emotional pain, leading to avoidance of treatment and worsening health risks. Socio-economic factors further complicate access to care, as clients worry about being judged, impacting their health outcomes. Additionally, many participants reported depression and confusion after their diagnosis, especially when they view their condition as terminal. This emphasis the need for comprehensive support that addresses both medical and psychological. Some STI clients feel hopeless. Nevertheless, the findings call for the need to integrate mental health services into STI care. In addition, deliberate public awareness programmes regarding STIs and their complications on mental well-being are crucial.
